# Supplementary material for: Stepwise Method and Factor Scoring in Multiple Regression Analysis of Cashmere Production in Liaoning Cashmere Goats
Source: Animals (Basel). 2022 Jul 23;12(15):1886. doi: 10.3390/ani12151886 (PMC9331259; doi:10.3390/ani12151886)
Supplement: Supplementary file 1 [file animals-12-01886-s001.zip › animals-1764694-supplementary.pdf]

Table S1. F test statistic.

Traits	Mean Square	F	P
**BSL（X_1）**	2123.790	2.324	0.000
**BH（X_2）**	2063.358	2.464	0.000
**CC（X_3）**	2604.759	4.023	0.000
**PC（X_4）**	10411.811	13.010	0.000
**CD（X_5）**	2244.017	2.744	0.000
**CW（X_6）**	2652.791	3.618	0.000
**HB（X_7）**	4597.651	9.835	0.000
**BW（X_8）**	2001.650	3.888	0.000

Note: F：F text, P：significant.
